# Effect of Chaihu Shugan Powder-Contained Serum on Glutamate-Induced Autophagy of Interstitial Cells of Cajal in the Rat Gastric Antrum

**DOI:** 10.1155/2019/7318616

**Published:** 2019-05-29

**Authors:** Ren-Qian Tan, Zhi Zhang, Jing Ju, Jiang-Hong Ling

**Affiliations:** ^1^The First Affiliated Hospital, Guangxi Medical University, Nanning 530021, China; ^2^Shuguang Affiliated Hospital, Shanghai University of Traditional Chinese Medicine, Shanghai 200021, China

## Abstract

Gastrointestinal (GI) motility disorder is caused by excessive autophagy of the interstitial cells of Cajal (ICC). Chaihu Shugan Powder (CSP) is a traditional Chinese medicine with therapeutic benefits in GI motility disorders; however, the underlying mechanism of its therapeutic effect in GI disorders, especially autophagy of ICC, remains unclear. Thus, this study investigated the effects of CSP-contained serum on glutamate-induced autophagy in rat gastric ICC, exploring its underlying mechanism. In vitro cultured rat stomach ICC were identified by fluorescence microscopy and then stimulated with glutamate (5 mmol/L) for 3 h to establish the autophagy model. These cells were then treated with 10% CSP-containing serum or the autophagy inhibitor 3-methyladenine (3-MA; 5 mmol/L) for 24 h. The control group was cultured with only 10% serum containing physiological saline. The viability of ICC was measured by the CCK-8 assay. The ultrastructure and autophagosomes of ICC were observed using transmission electron microscopy. LC3 expression was detected by immunofluorescence, and LC3, Beclin1, Bcl2, and PI3KC3 expression was detected by western blot analysis. Transmission electron microscopy showed abundant endoplasmic reticulum, mitochondria, and other organelles in the control group, whereas the cells in the autophagy model control group had clear autophagic vacuoles, which were not apparent in both CSP and 3-MA groups. ICC viability was significantly increased by CSP and 3-MA interventions (P < 0.01), accompanied by a decrease in LC3 fluorescence (P < 0.01). Moreover, the expression levels of LC3II/I, Beclin1, and PI3KC3 were significantly decreased (all P < 0.01) with CSP and 3-MA treatment, while Bcl2 expression level was higher than that of the model group (P < 0.01). Thus, CSP can reduce autophagic damage by enhancing Bcl2 expression and downregulating the expression of LC3, Beclin1, and PI3KC3 to protect ICC. These results highlight the potential of CSP in the treatment of GI motility disorders.

## 1. Introduction

Interstitial cells of Cajal (ICC) have been widely recognized as pacemakers and regulators of gastrointestinal (GI) motility and have therefore become the focus of research into the mechanism of GI motility disorders. Changes in the structure and number of ICC can often lead to GI function disorders, including idiopathic achalasia, diabetic gastropathy, chronic idiopathic intestinal pseudoobstruction, constipation, and functional dyspepsia [1–6]. Although the precise mechanism linking ICC changes with GI motility disorders has not yet been elucidated, our previous study [7] showed that excessive autophagy can lead to changes in the number and morphology of ICC. Autophagy is a highly conserved cellular protection process that is widely present in eukaryotic cells, involving the formation of autophagic vacuoles by the lysosomal pathway, thereby eradicating the cell's own senescent and damaged organelles or misfolded proteins to maintain homeostasis of the cellular environment [8]. However, uncontrolled autophagy of ICC can lead to slow-wave potentials, contraction rhythms of the smooth muscle, and loss of regulation in the transfer of neurotransmitters, contributing to GI motility disorders. Therefore, we hypothesized that GI motility disorder could be prevented by regulating the autophagy level of ICC [9].

Chaihu Shugan Powder (CSP) is a classical and effective prescription of traditional Chinese medicine (TCM), with strong and abundant evidence for its good therapeutic effects on GI motility disorders [10–14]. CSP was first described in the book “*Jing Yue Quan Shu*” written by Zhang Jiebin during the time of the Ming Dynasty. It consists of the following seven Chinese herbs:* Radix Bupleuri, Aurantii nobilis pericarpium, Paeonia, Szechwan Lovage rhizome, Fructus Aurantii, Nutgrass Galingale rhizome, and Glycyrrhiza uralensis*, traditionally administered with a dose ratio of 4:4:3:3:3:3:1. According to TCM theory, CSP effects include soothing stagnated Gan-qi, relieving the liver, regulating Qi, and relieving pain. CSP has been used to relieve bloating, abdominal pain, belching, anorexia, nausea, bitterness, acid reflux, constipation, and sloppy stool. The ingredients of the CSP decoction are mainly* albiflorin, ferulic acid, paeoniflorin, liquiritin, isoliquiritin, isoliquiritigenin, narirutin, naringin, naringenin, hesperidin, hesperetin,neohesperidinn, glycyrrhizic acid, alpha-cyperone, and 18-beta-glycyrrhizic acid *[15]. Zhang et al. [16] reported that CSP and its absorbed compound ferulic acid (FA) induced prokinetics via inhibiting serotonin, regulating the hypothalamic-pituitary axis, while increasing ghrelin and stimulating jejunal contraction simultaneously. Our previous study [17] showed that CSP can inhibit excessive autophagy of gastric antrum ICC in functional dyspepsia rats, thus promoting gastrointestinal motility. However, the efficacy of CSP in the treatment of GI motility disorders and the underlying mechanism are unknown. The application and popularization of drug-contained serum technology have broadened the research field of the effects of Chinese medicine compounds at the cellular level [18–20]. Bochu et al. [21] suggested the use of drug-contained serum in an isolated reaction system is suitable for investigations of pharmacological mechanisms, since this serum can not only prevent the physical and chemical properties of crude drug preparations from interfering with in vitro experiments but also can reflect the digestion and absorption of the Chinese medicine in the GI tract, and its subsequent biotransformation, thereby better reflecting the process that produces pharmacological effects. Therefore, the aim of the present study was to examine the effects of a CSP-contained serum on autophagy in the ICC of rats and to explore the underlying pharmacological mechanism in relation to autophagy.

## 2. Materials and Methods

### 2.1. Animals

The research was conducted according to protocols approved by the Experimental Animal Ethics Committee of Guangxi Medical University (No. 201611017) and was conducted in compliance with the Care and Use of Laboratory Animals guideline published by the US National Institutes of Health (NIH; publication No. 85-23; 1996). All efforts were made to minimize animal suffering.

Sprague-Dawley rats were obtained from Experimental Animal Center of Guangxi Medical University (Guang'xi, China; License no. SCXK (Gui) 2014-0002; Guang'xi, China) and randomly divided into two groups: group A (n = 20, 10 males and 10 females, 250 ± 20 g), which were used to prepare the drug-contained serum, and group B (n = 20, 10 males and 10 females, 150 ± 20 g), which were used for the isolation of ICC. All rats were acclimated in cages for three days prior to the experiments and maintained under a 12 h/12 h light cycle at 21 ± 2°C and 50 ± 5% relative humidity, with unlimited access to standard food and water.

### 2.2. Preparation of Drug-Contained Serum

CSP was prepared according to The People's Republic of China Pharmacopeia 1st Volume conventional dosage as follows: 6 g* Radix Bupleuri*, 4.5 g* Paeonia*, 4.5 g* Szechwan Lovage rhizome*, 4.5 g* Fructus Aurantii*, 6 g* Aurantii nobilis pericarpium*, 4.5 g* Nutgrass Galingale rhizome*, and 1.5 g* Glycyrrhiza uralensis*; all component herbs were purchased from the pharmacy of The First Affiliated Hospital of Guangxi Medical University. For the experiments, CSP was used at 10 times the normal recommended dose for a 60-kg adult (according to the Chinese Medicine book* Jing Yue Quan Shu*, based on the surface area conversion algorithm for the standard rat body [22] and was concentrated to 105% (1.05 g/mL).

Rats of group A were randomly divided into two groups and were intragastrically administered with physiological saline and the CSP decoction, respectively, at a concentration of 1.5 mL/100 g twice daily at 12 h intervals for three consecutive days. Under anesthesia, blood was extracted from the abdominal aorta 1h after the last administration, and the serum was collected after standing and centrifugation. The serum was then inactivated in water (56°C, 0.5 h), filtered, sterilized by a 0.22 *μ*m microporous membrane filter, and stored at -80°C until use. At the end of the experiment, all rats were sacrificed by cervical dislocation.

### 2.3. ICC Isolation and Culture

The 20 rats in group B were euthanized by cervical dislocation. The stomachs were isolated and the antrum was dissected out for subsequent use. After longitudinal laparotomy, the antrum was immediately dissected, opened along the lesser curvature of the stomach, and rinsed free of content with ice-cold D-Hanks solution. The gastric mucosa was carefully peeled away from the smooth muscle layers with sharp forceps, and then cut into small pieces of approximately 1 × 1 mm. The pieces were then incubated in a Ca^2+^-free dispersal solution containing collagenase type II (1.3 mg/mL; Sigma, USA) for approximately 50 min at 37°C. Single cells were obtained by gentle agitation for 5 min with a wide-bore pipette, and the dispersed cells were filtered through 200 *μ*m mesh filters. The dispersed cells were plated onto a 25 mm diameter culture flask and cultured at 37°C in a 95% O_2_-5% CO_2_ incubator in ICC growth medium (M199; Hyclone, USA) supplemented with 2% antibiotics and 25 ng/mL murine stem cell factor (Sigma, USA). ICC were identified immunologically by incubation with rabbit anti-rat c-Kit monoclonal antibody (Pierce, USA) at a dilution of 1:200 for 60 min, which allowed for differentiation of ICC from other cell types in the culture based on morphological differences detected by light and fluorescence microscopy.

### 2.4. Autophagy Model Establishment and Treatments

The autophagy model was established according to recent methods reported by Tan et al. [23] using incubation with 5 mol/L glutamate (L-glutamic acid; Sigma, USA) for 3 h. For comparison, the control group was cultured with 10% serum containing physiological saline. The cells in the autophagy group were then treated with or without 10% serum containing CSP or 5 mol/L 3-methyladenine (3-MA), an autophagy inhibitor.

### 2.5. Cell Viability

The viability of ICC in the different groups was assessed with Cell Counting Kit-8 (CCK-8; Dojindo, Japan). In brief, ICC were seeded at a density of 1 × 10^4^ cells/well in 96-well flat-bottomed microplates. Once the cells attached to the bottom of the culture vessel, the culture medium was removed from each well. Different concentrations of the respective culture medium for each group were added to the wells, and the cells were cultured for 24 h. CCK-8 reagent (10 *μ*L) was added to each well of a 96-well flat-bottomed microplate that contained 100 *μ*L of culture medium to reach a final concentration of 10 *μ*L/100 *μ*L and incubated for an additional 4 h at 37°C. The absorbance rate was measured at 450 nm on an auto microplate reader (Multiskan FC, Thermo, USA). All experiments were conducted in triplicate on six separate occasions.

### 2.6. Transmission Electron Microscopy

After centrifugation, the cells were fixed for 2 h with 3% glutaraldehyde in phosphate buffered saline (PBS, pH 7.2). After rinsing with PBS, the samples were post-fixed in 1% osmium tetroxide for 1 h at 4°C, dehydrated in a graded series of acetone, and embedded in Eponate 812 resin. Ultrathin sections were cut into 50–70 nm thick slices, double-stained with uranyl acetate and lead citrate, and observed on a Hitachi (Japan) transmission electron microscope.

### 2.7. Immunofluorescence

ICC in the logarithmic growth phase was plated onto sterile glass coverslips in a 24-well flat-bottomed microplate at a density of 5 × 10^3^ cells/well. Once the cells attached to the bottom of the culture vessel, ICC was cultured according to the group requirements mentioned above. The culture medium was removed from the coverslips, all of the cultured cells were fixed with ice-cold paraformaldehyde for 10 min and then washed in PBS three times for 5 min each. The samples were incubated in 5% nonfat milk for 10 min at room temperature to reduce nonspecific staining. After incubation with an anti-LC3 antibody (1:100; Novus, USA) at 4°C overnight; the samples were washed in PBS three times for 10 min each. Immunoreactivity was detected with a fluorescein isothiocyanate-conjugated secondary antibody (anti-rabbit IgG, 1:200) in the dark for 30 min at 37°C. The nuclei were labeled with 4′,6-diamidino-2-phenylindole (Beyotime, China). The sections were examined by fluorescence microscopy (Upright fluorescence microscope; Nikon, Japan), respectively.

### 2.8. Western Blot

The cells were washed with ice-cold PBS and lysed with ice-cold lysis buffer containing a protease inhibitor cocktail. The protein samples were harvested, resolved on sodium dodecyl sulfate-polyacrylamide gel electrophoresis gels, and transferred to polyvinylidene difluoride membranes. After blocking with 5% nonfat milk for 2 h, the membranes were incubated overnight at 4°C with the following primary antibodies: LC3 (1:1000; Novus, USA), Beclin1 (1:1000; Novus, USA), Bcl2 (1:1000; CST, USA), PI3KC3 (1:1000; CST, USA), and GAPDH (1:1000; SAB, USA). After washing in PBS (supplemented with 0.1% Tween 20) three times for 5 min each, the samples were incubated with the appropriate secondary antibody and developed with enhanced chemiluminescence.

### 2.9. Statistical Analyses

Data are presented as mean ± standard deviation. Differences between groups were assessed using one-way analysis of variance followed by Tukey's test with SPSS 23.0 software. All statistical tests were two-tailed, and a* P* value < 0.05 was considered statistically significant.

## 3. Results

### 3.1. ICC Viability

**Figure 1:**
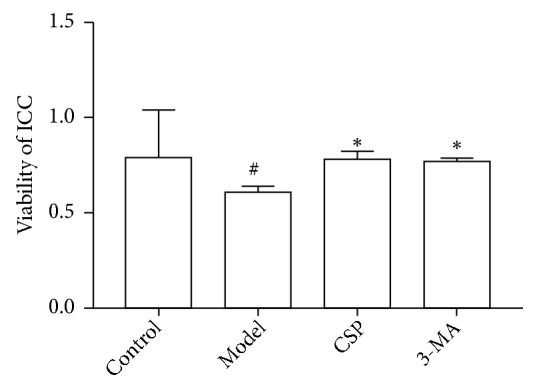
The viability of interstitial cells of Cajal (ICC) under glutamate-induced autophagy with and without treatment of the known autophagy inhibitor, 3-MA, or Chaihu Shugan powder (CSP). ^#^*P* < 0.01 versus control group; ^*∗*^*P* < 0.05 versus model group (N = 6 per group).

The CCK-8 assay demonstrated that the OD value of the model group significantly decreased compared to that of the control group (*P*< 0.001), whereas the OD values of the CSP and 3-MA groups were significantly increased compared to that of the model group (both* P*< 0.001) (Figure 1).

### 3.2. Ultrastructure of ICC

**Figure 2:**
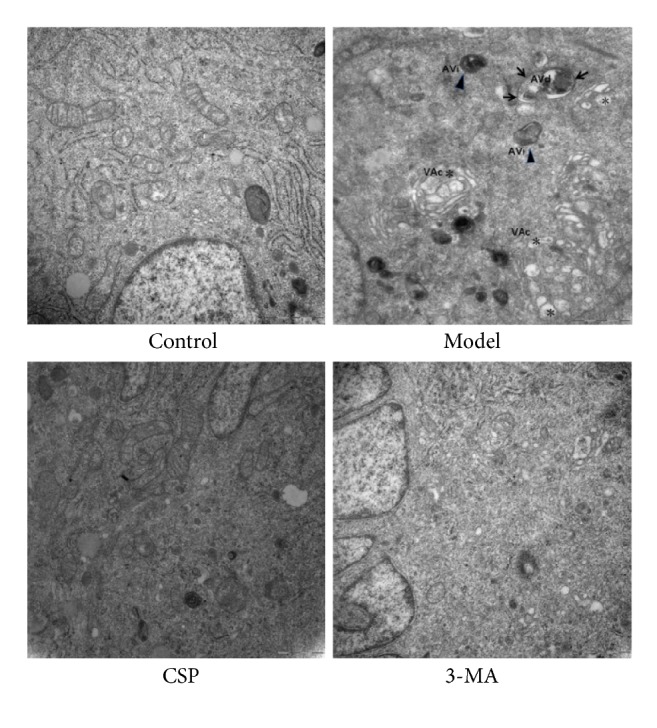
Evidence of autophagy and the ultrastructure of interstitial cells of Cajal (ICC) observed under transmission electron microscopy (×30, 000). ▲: initial autophagic vacuoles, AVi; →: degrading autophagic vacuoles, and AVd; *∗*: vacuole, VAc.

Transmission electron microscopy revealed that ICC in the control group contained abundant organelles, such as endoplasmic reticulum and mitochondria, without obvious autophagosomes and autophagic vacuoles. There was an apparent number of autophagic vacuoles and vacuoles in the model group than in the normal control group, suggesting autophagy in ICC. Interestingly, CSP and 3-MA treatment significantly inhibited autophagy (Figure 2).

### 3.3. Protein Expression of Autophagy Markers

**Figure 3:**
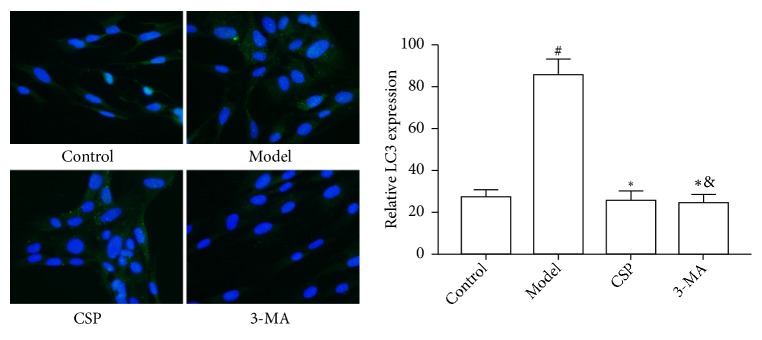
Observation of LC3 in the interstitial cells of Cajal (ICC) of each group under fluorescence microscopy (×200). ^&^*P *< 0.05 versus control; ^#^*P *< 0.01 versus control; *∗P *< 0.01 versus model. (N = 15 per group).

**Figure 4:**
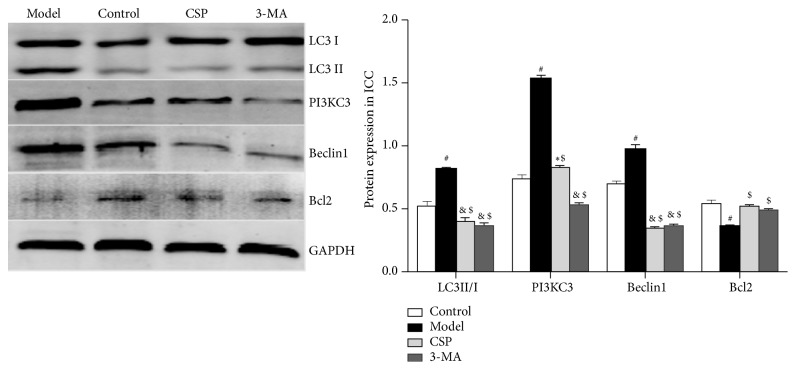
LC3, PI3KC3, Beclin1, and Bcl2 protein expression in interstitial cells of Cajal (ICC). The expression levels were normalized to that of the housekeeping protein GAPDH. ^&^*P *< 0.05 versus control; ^#^*P *< 0.01 versus control; ^$^*P* < 0.01 versus model; ^*∗*^*P* < 0.05 versus 3-MA (N = 3 per group).

Compared with the control group, the fluorescent intensity of LC3 in the ICC of the model group was significantly increased (*P*< 0.01), whereas the LC3 fluorescence of the CSP and 3-MA groups was significantly lower than that of the model group (*P*< 0.05) (Figure 3). This finding was further confirmed by western blot analysis (Figure 4), which showed that there was a significant increase in the levels of LC3, PI3KC3, and Beclin1, but decrease in Bcl2 protein expression in the model group (*P*< 0.05). However, the CSP and 3-MA treatment markedly reversed the abnormal changes of these proteins (*P*< 0.05), suggesting that CSP can reduce autophagy in ICC.

### 3.4. Discussion

Recently, ICC have emerged as the basic determinant of GI motility, and thus serve as an ideal tool for investigating the mechanisms of GI motility and determining treatment strategies for related disorders [24, 25]. According to the theory of TCM, the liver controls conveyance and dispersion, loving rising, and smooth, hating depression. Presence of external pathogens, emotional disorders, dietary irregularities, and spleen-stomach weakness lead to liver dysfunction. Additionally, the pattern of liver qi invading the stomach, leading to spleen and stomach dysfunction is considered to be the main cause of GI motility disorders. CSP is a classical and effective prescription representative of the Shugan Qi method. It can soothe the liver and regulate qi, move qi to relieve pain, promote digestion, and remove food stagnation that can further relieve clinical symptoms of GI motility disorder and eventually restore normal digestive function. Although CSP has been used as an effective prokinetic for GI motility disorders in TCM [16, 26], the underlying mechanism of its effects remains unclear so far. Here, we demonstrate a clear attenuating effect of CSP on excessive autophagy of ICC that could contribute to dysregulated GI motility.

Autophagy is a biological process through which cells maintain their homeostasis and physiology [27]. Under stress, autophagy is responsible for the removal of damaged organelles, production of energy for cells, and maintenance of internal environment stability. However, excessive autophagy can induce cell death. LC3 is a homolog of yeast autophagy-associated protein 8 (Atg8) and is essential for autophagy. It is involved in the formation of autophagic vesicles and therefore is an important indicator of autophagic activity [28]. LC3II is a widely used autophagy marker; when autophagy occurs in cells, LC3I, which mainly exists in the cytoplasm, is ubiquitin-modified and converts to the LC3II form with phosphatidyl ethanolamine on the surface of the autophagic foam membrane. Its content is proportional to the number of autophagic vacuoles. By examining LC3II/LC3I, we can determine the number of autophagosomes and infer the strength of autophagy [29].

Autophagic activity is regulated by many signaling pathways, and the combination of PI3KC3 and Beclin1 to form autophagosomes is the main positive regulatory mechanism. PI3KC3 is a homolog of Vps34, which phosphorylates the third site protein of phosphatidylinositol in eukaryotes to form a complex with Beclin1. siRNA silences the expression of PI3KC3 and attenuates the expression of autophagy [30]. Beclin1 is a homologous gene of yeast ATG6 and is also the most important regulator of the positive regulatory mechanism [31]. Absence of Beclin1 weakens autophagic activity. The reintroduction of Beclin1 can effectively regulate autophagy pathway and enhance cell death metabolism [32]. Beclin1 interacts with many proteins to form multiple functionally distinct complexes [33]. Through the formation of complexes with PI3KC3, Beclin1 regulates the localization of autophagy precursors and in turn regulates autophagic activity [34]. Contrastingly, Bcl2 acts as an inhibitor of autophagy and apoptosis and protects cells [35, 36]. Although upregulated Beclin1 expression in mammalian cells can induce autophagy [37], Bcl2 can inhibit the Beclin1-dependent autophagy by forming a complex with Beclin1. Thus, Beclin1 is a key factor in regulating and maintaining the balance between autophagy and apoptosis in cells; when Beclin1 forms a complex with PI3KC3, it activates the process of autophagy, and when Beclin1 forms a complex with Bcl2, it activates apoptosis.

Glutamate is an important amino acid that maintains normal physiological functions of the body and is involved in the synthesis of various proteins. Oudenhove et al. [38] suggested that an excess amount of glutamate could induce neuronal cell autophagy due to its excitotoxic and oxidative stress impairment. Chen et al. [39] suggested that glutamate could induce the formation and recruitment of PI3KC3 and Beclin1, form complexes, and participate in the formation of autophagic membranes. However, the glutamate-induced activation of autophagy in ICC is not clear. In the present study, glutamate treatment markedly reduced the viability of rat ICC and increased the number of autophagic vacuoles. Additionally, increased expression of autophagy markers such as LC3, Beclin1, and PI3KC3 and reduced expression of the apoptosis regulator protein Bcl2 was observed. This indicated that the glutamate-mediated induction of autophagy was successful. Furthermore, treatment with the known autophagy inhibitor 3-MA inhibited autophagy and the same effect was also detected for CSP. Thus, our study suggests that this mechanism might involve increasing the levels of autophagy-related proteins while simultaneously inhibiting the proteins that negatively regulate autophagy.

Importantly, both CSP and 3-MA could improve the autophagic damage induced by glutamate. Klionsky et al. [40] suggested that the mechanism by which 3-MA inhibits autophagy may involve suppressing the expression of PI3KC3 and thereby preventing the formation of autophagosomes. Chen et al. [41] also found that 3-MA could inhibit autophagy by reducing the expression of Beclin1. Therefore, our findings suggest that CSP may inhibit autophagy in the same manner as 3-MA.

Collectively, these results highlight the protective effect of CSP on excessive autophagy of ICC by reducing the high expression of LC3, Beclin1, and PI3KC3 proteins and enhancing the low expression of Bcl2.

### 3.5. Conclusions

Our data suggest that CSP shows significant gastroprokinetic effects by inhibiting excessive autophagy via prevention of Bcl2-Beclin1 complex dissociation.

These effects highlight CSP as a strong candidate for the treatment of GI motility disorders. However, the effects induced by TCM are currently difficult to reproduce because of limited information available regarding the absorbed bioactive compounds. To resolve this issue and develop a novel prokinetic strategy based on CSP, further research is required to identify the specific compounds responsible for the inhibitory effects of CSP and to determine their underlying mechanisms of action.

## Data Availability

The datasets used and/or analyzed during the current study are available from the corresponding author on reasonable request.

## List of Abbreviations

3-MA: 3-Methyladenine
CSP: Chaihu Shugan powder
TCM: Traditional Chinese medicine
CCK-8: Cell Counting Kit-8
ICC: Interstitial cells of Cajal;
GI: Gastrointestinal
PBS: Phosphate-buffered saline.

## References

[1] Pardi D. S., Miller S. M., Miller D. L. (2002). Paraneoplastic dysmotility: loss of interstitial cells of Cajal. *American Journal of Gastroenterology*.

[2] Nakahara M., Isozaki K., Hirota S. (2002). Deficiency of KIT-positive cells in the colon of patients with diabetes mellitus. *Journal of Gastroenterology and Hepatology*.

[3] Streutker C. J., Huizinga J. D., Campbell F., Ho J., Riddell R. H. (2003). Loss of CD117 (c-kit)- and CD34-positive ICC and associated CD34-positive fibroblasts defines a subpopulation of chronic intestinal pseudo-obstruction. *The American Journal of Surgical Pathology*.

[4] Villanacci V., Annese V., Cuttitta A. (2010). An immunohistochemical study of the myenteric plexus in idiopathic achalasia. *Journal of Clinical Gastroenterology*.

[5] Yin J., Chen J. D. Z. (2008). Roles of interstitial cells of Cajal in regulating gastrointestinal motility: In vitro versus in vivo studies. *Journal of Cellular and Molecular Medicine*.

[6] Sanders K. M., Ördög T., Ward S. M. (2002). Physiology and pathophysiology of the interstitial cells of Cajal: From bench to bedside. IV. Genetic and animal models of GI motility disorders caused by loss of interstitial cells of Cajal. *American Journal of Physiology-Gastrointestinal and Liver Physiology*.

[7] Zhang L., Zeng L., Deng J. (2018). Investigation of autophagy and differentiation of myenteric interstitial cells of Cajal in the pathogenesis of gastric motility disorders in rats with functional dyspepsia. *Biotechnology and Applied Biochemistry*.

[8] Watanabe T., Kuma A., Mizushima N. (2011). Physiological role of autophagy in metabolism and its regulation mechanism. *Nihon Rinsho. Japanese Journal of Clinical Medicine*.

[9] Ji Y. X., Ling J. H., Pang L. M., Huang G. H., Jiang L., Zheng C. W. (2013). The interstitial cells of Cajal, autophagy and gastrointestinal motility disorders. *Journal of Guangxi Medical University*.

[10] Su X. Y., Chen Y. Y. (2016). Treatment of functional dyspepsia with Chaihu Shugan powder: a meta-analysis. *Chinese Journal of Integrated Traditional and Western Medicine*.

[11] Chen W. Q., Yang H. G. (2003). Efficacy of modified Chaihu Shugan powder on 61 cases with irritable bowel syndrome. *New Journal of Traditional Chinese Medicine*.

[12] Qiao C. A. (2010). Clinical study of modified Chaihu Shugan powder on functional dyspepsia. *Journal of Sichuan Traditional Chinese Medicine*.

[13] Cui Q. Z., Wang X. Y. (2008). Treatment of 54 cases of constipation with Chaihu Shugan powder. *China Journal of Traditional Chinese Medicine and Pharmacy*.

[14] Yang N., Jiang X., Qiu X., Hu Z., Wang L., Song M. (2013). Modified Chaihu Shugan powder for functional dyspepsia: meta-analysis for randomized controlled trial. *Evidence-Based Complementary and Alternative Medicine*.

[15] Fan R., Huang X., Wang Y. (2012). Ethnopharmacokinetic-and activity-guided isolation of a new antidepressive compound from Fructus Aurantii found in the traditional chinese medicine Chaihu-Shugan-San: a new approach and its application. *Evidence-Based Complementary and Alternative Medicine*.

[16] Zhang Y.-J., Huang X., Wang Y. (2011). Ferulic acid-induced anti-depression and prokinetics similar to Chaihu-Shugan-San via polypharmacology. *Brain Research Bulletin*.

[17] Zeng L. J., Ling J. H., Deng J., Wang Y. J., Zhang Z., Lei Z. J. (2017). Effect of Chaihu Shugan powder on autophagy of interstitial cells of interstitial Cajal in gastric antrum of rats with functional dyspepsia. *Lishizhen Medicine and Materia Medica Research*.

[18] Liu Z. Q., Yin D. k., Han L. (2013). Protective effect of medicated serum prepared with Taohong Siwu Tang on hydrogen peroxide-induced human umbilical vein endothelial cells. *Zhongguo Zhong Yao Za Zhi*.

[19] Guo X., Li M. L., Liu X. L., Jia B., Shi H. X., Shen T. (2015). Effect of aconite containing serum on L-type calcium channel in rat ventricular myocytes. *Chinese Journal of Experimental Traditional Medical Formulae*.

[20] Xu S., Dong Y., Yang K., Gong XY., Li N. (2016). Effect of Sanzi Yangqin Tang on CysLTs mediated inflammatory pathways in bronchial epithelial cells. *Chinese Journal of Experimental Traditional Medical Formulae*.

[21] Bochu W., Liancai Z., Qi C. (2005). Primary study on the application of Serum Pharmacology in Chinese traditional medicine. *Colloids and Surfaces B: Biointerfaces*.

[22] Xu S. Y., Bian R. L., Chen X. (2002). *Experimental Methodology of Pharmacology*.

[23] Tan R. Q., Zhang Z., Ning H. E., Zhang L. M., Wang Y., Ling J. H. (2018). Establishment of autophagy model of rat gastric interstitial cells of Cajal induced by glutamic acid. *Chinese Journal of Pathophysiology*.

[24] Ward S. M., Burns A. J., Torihashi S., Sanders K. M. (1994). Mutation of the proto‐oncogene c‐kit blocks development of interstitial cells and electrical rhythmicity in murine intestine. *The Journal of Physiology*.

[25] Sanders K. M. (1996). A case for interstitial cells of Cajal as pacemakers and mediators of neurotransmission in the gastrointestinal tract. *Gastroenterology*.

[26] Li Y.-H., Zhang C.-H., Wang S.-E., Qiu J., Hu S.-Y., Xiao G.-L. (2009). Effects of Chaihu Shugan San on behavior and plasma levels of corticotropin releasing hormone and adrenocorticotropic hormone of rats with chronic mild unpredicted stress depression. *Journal of Chinese Integrative Medicine*.

[27] Tuloup-Minguez V., Hamaï A., Greffard A., Nicolas V., Codogno P., Botti J. (2013). Autophagy modulates cell migration and *β*1 integrin membrane recycling. *Cell Cycle*.

[28] Kabeya Y., Mizushima N., Ueno T. (2000). LC3, a mammalian homologue of yeast Apg8p, is localized in autophagosome membranes after processing. *EMBO Journal*.

[29] Cherra S. J., Kulich S. M., Uechi G. (2010). Regulation of the autophagy protein LC3 by phosphorylation. *The Journal of Cell Biology*.

[30] Juhász G., Hill J. H., Yan Y. (2008). The class III PI (3) K Vps34 promotes autophagy and endocytosis but not TOR signaling in Drosophila. *The Journal of Cell Biology*.

[31] He C., Levine B. (2010). The Beclin 1 interactome. *Current Opinion in Cell Biology*.

[32] Shin J. Y., Hong S.-H., Kang B., Minai-Tehrani A., Cho M.-H. (2013). Overexpression of beclin1 induced autophagy and apoptosis in lungs of K-rasLA1 mice. *Lung Cancer*.

[33] Maiuri M. C., Criollo A., Kroemer G. (2010). Crosstalk between apoptosis and autophagy within the Beclin 1 interactome. *EMBO Journal*.

[34] Scarlatti F., Maffei R., Beau I., Codogno P., Ghidoni R. (2008). Role of non-canonical Beclin 1-independent autophagy in cell death induced by resveratrol in human breast cancer cells. *Cell Death & Differentiation*.

[35] Strappazzon F., Vietri-Rudan M., Campello S. (2011). Mitochondrial BCL-2 inhibits AMBRA1-induced autophagy. *EMBO Journal*.

[36] Sun Z.-J., Zhang L., Hall B., Bian Y., Gutkind J. S., Kulkarni A. B. (2012). Chemopreventive and chemotherapeutic actions of mTOR inhibitor in genetically defined head and neck squamous cell carcinoma mouse model. *Clinical Cancer Research*.

[37] Liang X. H., Jackson S., Seaman M. (1999). Induction of autophagy and inhibition of tumorigenesis by beclin 1. *Nature*.

[38] Van Oudenhove L., Aziz Q. (2013). The role of psychosocial factors and psychiatric disorders in functional dyspepsia. *Nature Reviews Gastroenterology & Hepatology*.

[39] Chen Z. Y., Chen M. G., Lu T. T., Ni G. Z., Liu X. F., Xu G. L. (2011). Autophagy is activated during glutamicacid-induced cortical neuron injury. *Chinese Journal of Pathophysiology*.

[40] Klionsky D. J., Codogno P., Cuervo A. M. (2010). A comprehensive glossary of autophagy-related molecules and processes. *Autophagy*.

[41] Chen Z., Lu T., Yue X. (2010). Neuroprotective effect of ginsenoside Rb1 on glutamate-induced neurotoxicity: With emphasis on autophagy. *Neuroscience Letters*.

